# Supplementary figures and images for: Recovering a lost seismic disaster. The destruction of El Castillejo and the discovery of the earliest historic earthquake affecting the Granada region (Spain)
Source: PLoS One. 2024 Apr 17;19(4):e0300549. doi: 10.1371/journal.pone.0300549 (PMC11023601; doi:10.1371/journal.pone.0300549)

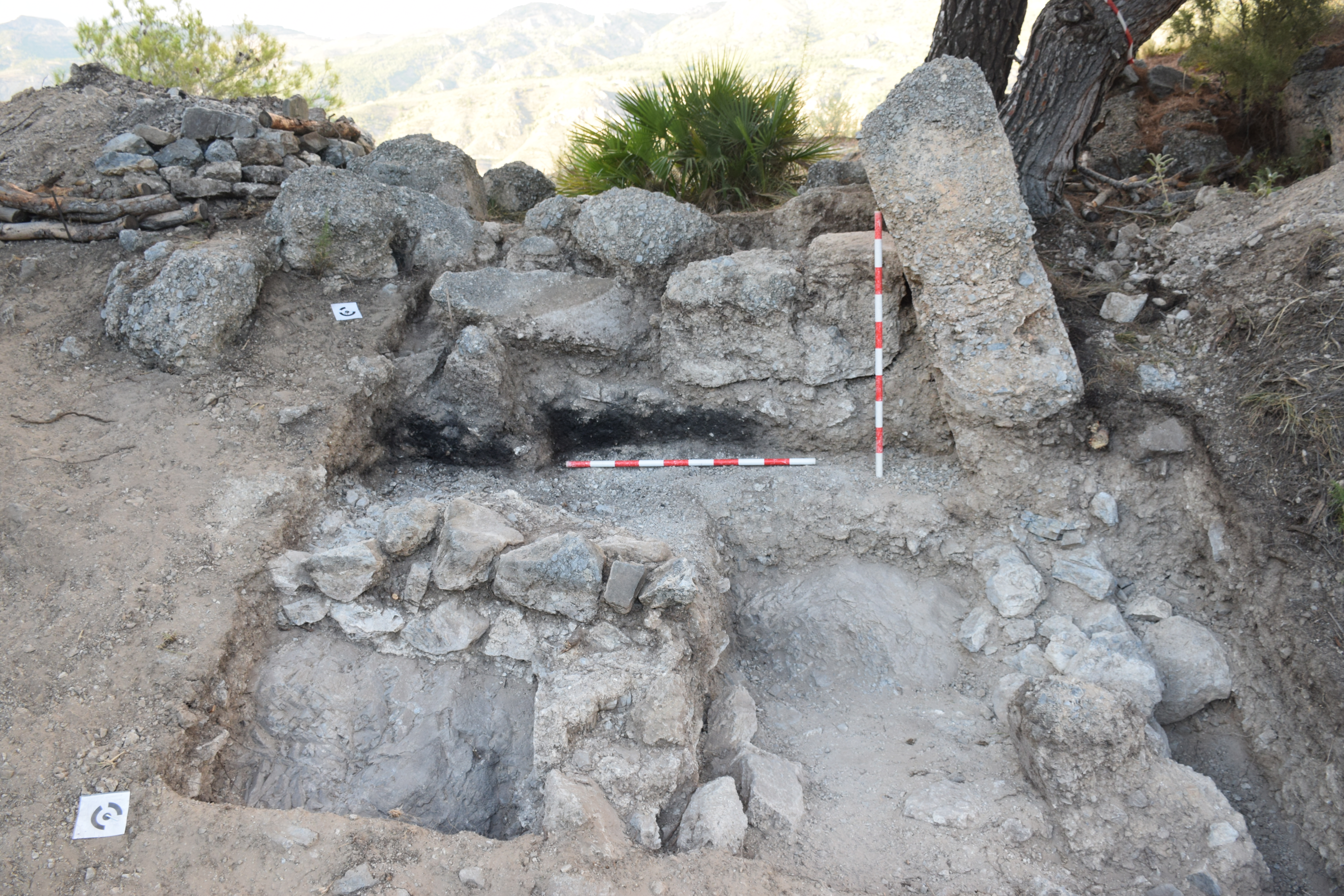

Supplement: S1 Fig — (JPG) [file pone.0300549.s001.JPG]
