# Supplementary material for: Recovering a lost seismic disaster. The destruction of El Castillejo and the discovery of the earliest historic earthquake affecting the Granada region (Spain)
Source: PLoS One. 2024 Apr 17;19(4):e0300549. doi: 10.1371/journal.pone.0300549 (PMC11023601; doi:10.1371/journal.pone.0300549)
Supplement: S1 File — (PDF) [file pone.0300549.s002.pdf]

Scottish Universities Environmental Research Centre
Rankine Avenue, Scottish Enterprise Technology Park, East Kilbride, Glasgow G75 0QF, Scotland, UK
Director: Professor F M Stuart Tel: +44 (0)1355 223332 Fax: +44 (0)1355 229898 www.glasgow.ac.uk/suerc

RADIOCARBON DATING CERTIFICATE
20 February 2019

**Laboratory Code**	SUERC-84641 (GU50187)
**Submitter**	Paolo Forlin<br>University of Durham<br>Department of Archaeology<br>South Road<br>DH1 3LE<br>Durham
**Site Reference**	GR-EC18
**Context Reference**	207
**Sample Reference**	C14-01
**Material**	Charcoal : Pinus sp.
**$\delta^{13}C$ relative to VPDB**	-25.3 ‰
**Radiocarbon Age BP**	782 ± 34

N.B. The above ^{14}C age is quoted in conventional years BP (before 1950 AD) and requires calibration to the calendar timescale. The error, expressed at the one sigma level of confidence, includes components from the counting statistics on the sample, modern reference standard and blank and the random machine error.

Samples with a SUERC coding are measured at the Scottish Universities Environmental Research Centre AMS Laboratory and should be quoted as such in any reports within the scientific literature. The laboratory GU coding should also be given in parentheses after the SUERC code.

Detailed descriptions of the methods employed by the SUERC Radiocarbon Laboratory can be found in Dunbar et al. (2016) *Radiocarbon 58(1) pp.9-23.*

For any queries relating to this certificate, the laboratory can be contacted at suerc-c14lab@glasgow.ac.uk.

Conventional age and calibration age ranges calculated by :

Checked and signed off by :

The University of Glasgow, charity number SC004401

The University of Edinburgh is a charitable body, registered in Scotland, with registration number SC005336

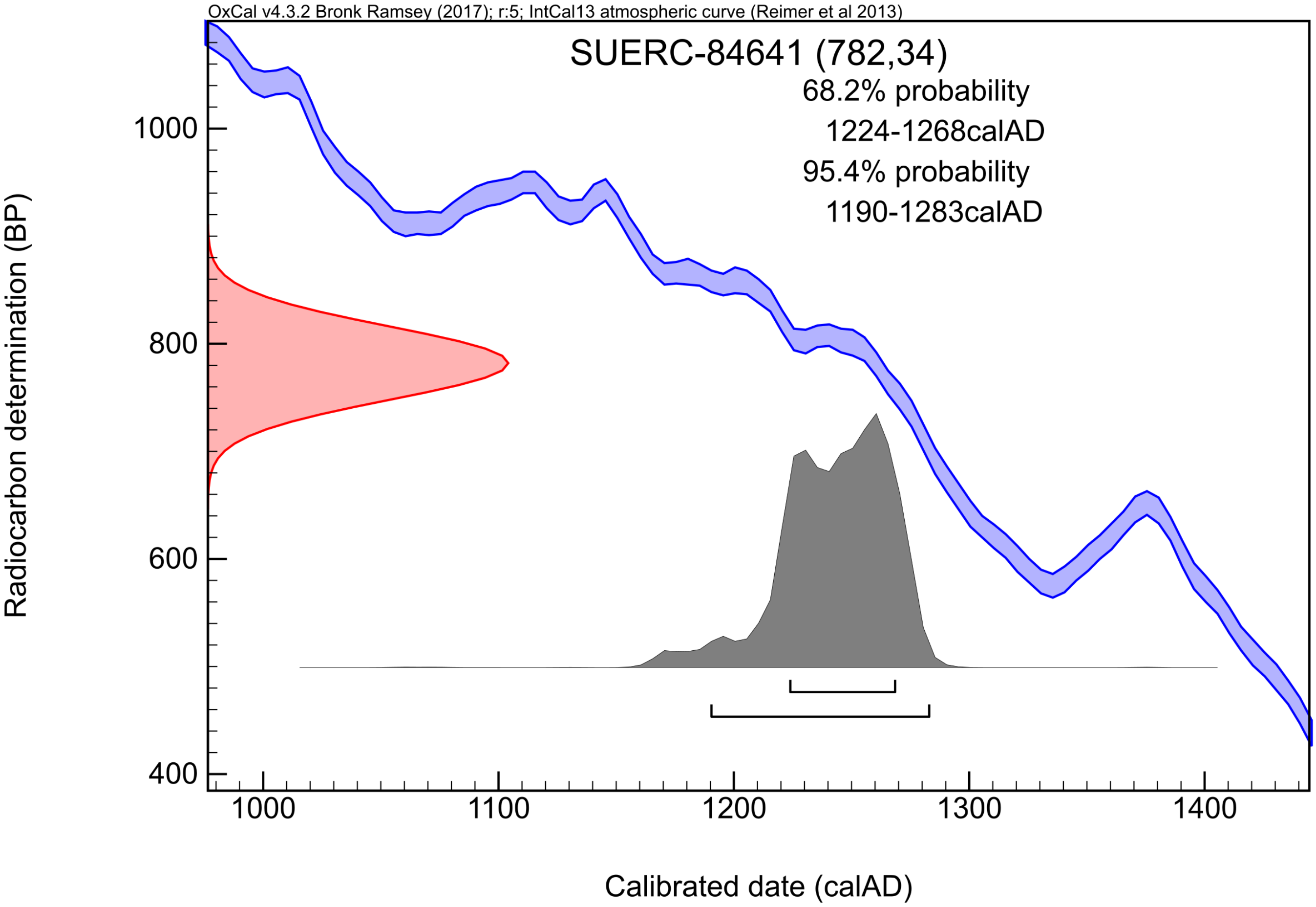

The radiocarbon age given overleaf is calibrated to the calendar timescale using the Oxford Radiocarbon Accelerator Unit calibration program OxCal 4.*

The above date ranges have been calibrated using the IntCal13 atmospheric calibration curve.†

Please contact the laboratory if you wish to discuss this further.

* Bronk Ramsey (2009) *Radiocarbon 51(1) pp.337-60*
† Reimer et al. (2013) *Radiocarbon 55(4) pp.1869-87*

Scottish Universities Environmental Research Centre
Rankine Avenue, Scottish Enterprise Technology Park, East Kilbride, Glasgow G75 0QF, Scotland, UK
Director: Professor F M Stuart Tel: +44 (0)1355 223332 Fax: +44 (0)1355 229898 www.glasgow.ac.uk/suerc

RADIOCARBON DATING CERTIFICATE
20 February 2019

**Laboratory Code**	SUERC-84642 (GU50188)
**Submitter**	Paolo Forlin<br>University of Durham<br>Department of Archaeology<br>South Road<br>DH1 3LE<br>Durham
**Site Reference**	GR-EC18
**Context Reference**	207
**Sample Reference**	C14-02
**Material**	Charcoal : Pinus sp.
**$\delta^{13}C$ relative to VPDB**	-25.0 ‰
**Radiocarbon Age BP**	800 ± 34

N.B. The above ^{14}C age is quoted in conventional years BP (before 1950 AD) and requires calibration to the calendar timescale. The error, expressed at the one sigma level of confidence, includes components from the counting statistics on the sample, modern reference standard and blank and the random machine error.

Samples with a SUERC coding are measured at the Scottish Universities Environmental Research Centre AMS Laboratory and should be quoted as such in any reports within the scientific literature. The laboratory GU coding should also be given in parentheses after the SUERC code.

Detailed descriptions of the methods employed by the SUERC Radiocarbon Laboratory can be found in Dunbar et al. (2016) *Radiocarbon 58(1) pp.9-23.*

For any queries relating to this certificate, the laboratory can be contacted at suerc-c14lab@glasgow.ac.uk.

Conventional age and calibration age ranges calculated by :

Checked and signed off by :

The University of Glasgow, charity number SC004401

The University of Edinburgh is a charitable body, registered in Scotland, with registration number SC005336

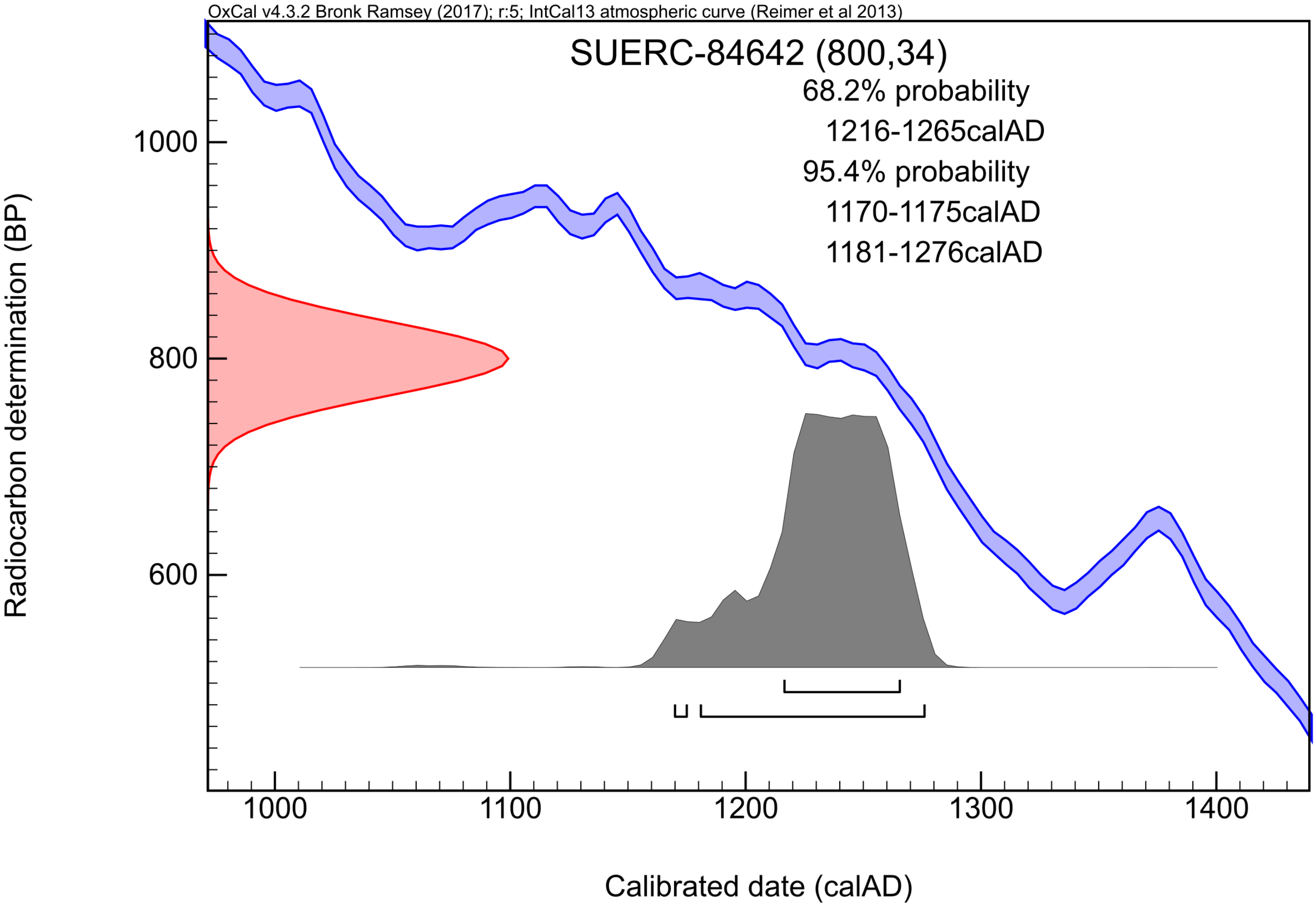

The radiocarbon age given overleaf is calibrated to the calendar timescale using the Oxford Radiocarbon Accelerator Unit calibration program OxCal 4.*

The above date ranges have been calibrated using the IntCal13 atmospheric calibration curve.†

Please contact the laboratory if you wish to discuss this further.

* Bronk Ramsey (2009) *Radiocarbon 51(1) pp.337-60*
† Reimer et al. (2013) *Radiocarbon 55(4) pp.1869-87*

Scottish Universities Environmental Research Centre
Rankine Avenue, Scottish Enterprise Technology Park, East Kilbride, Glasgow G75 0QF, Scotland, UK
Director: Professor F M Stuart   Tel: +44 (0)1355 223332   Fax: +44 (0)1355 229898   www.glasgow.ac.uk/suerc

RADIOCARBON DATING CERTIFICATE
20 February 2019

**Laboratory Code**	SUERC-84646 (GU50189)
**Submitter**	Paolo Forlin<br>University of Durham<br>Department of Archaeology<br>South Road<br>DH1 3LE<br>Durham
**Site Reference**	GR-EC18
**Context Reference**	210
**Sample Reference**	C14-03
**Material**	Charred plant remain : Hordeum sp.
**$\delta^{13}C$ relative to VPDB**	-23.9 ‰
**Radiocarbon Age BP**	816 ± 34

N.B. The above ^{14}C age is quoted in conventional years BP (before 1950 AD) and requires calibration to the calendar timescale. The error, expressed at the one sigma level of confidence, includes components from the counting statistics on the sample, modern reference standard and blank and the random machine error.

Samples with a SUERC coding are measured at the Scottish Universities Environmental Research Centre AMS Laboratory and should be quoted as such in any reports within the scientific literature. The laboratory GU coding should also be given in parentheses after the SUERC code.

Detailed descriptions of the methods employed by the SUERC Radiocarbon Laboratory can be found in Dunbar et al. (2016) *Radiocarbon 58(1) pp.9-23.*

For any queries relating to this certificate, the laboratory can be contacted at suerc-c14lab@glasgow.ac.uk.

Conventional age and calibration age ranges calculated by :

Checked and signed off by :

The University of Glasgow, charity number SC004401

The University of Edinburgh is a charitable body, registered in Scotland, with registration number SC005336

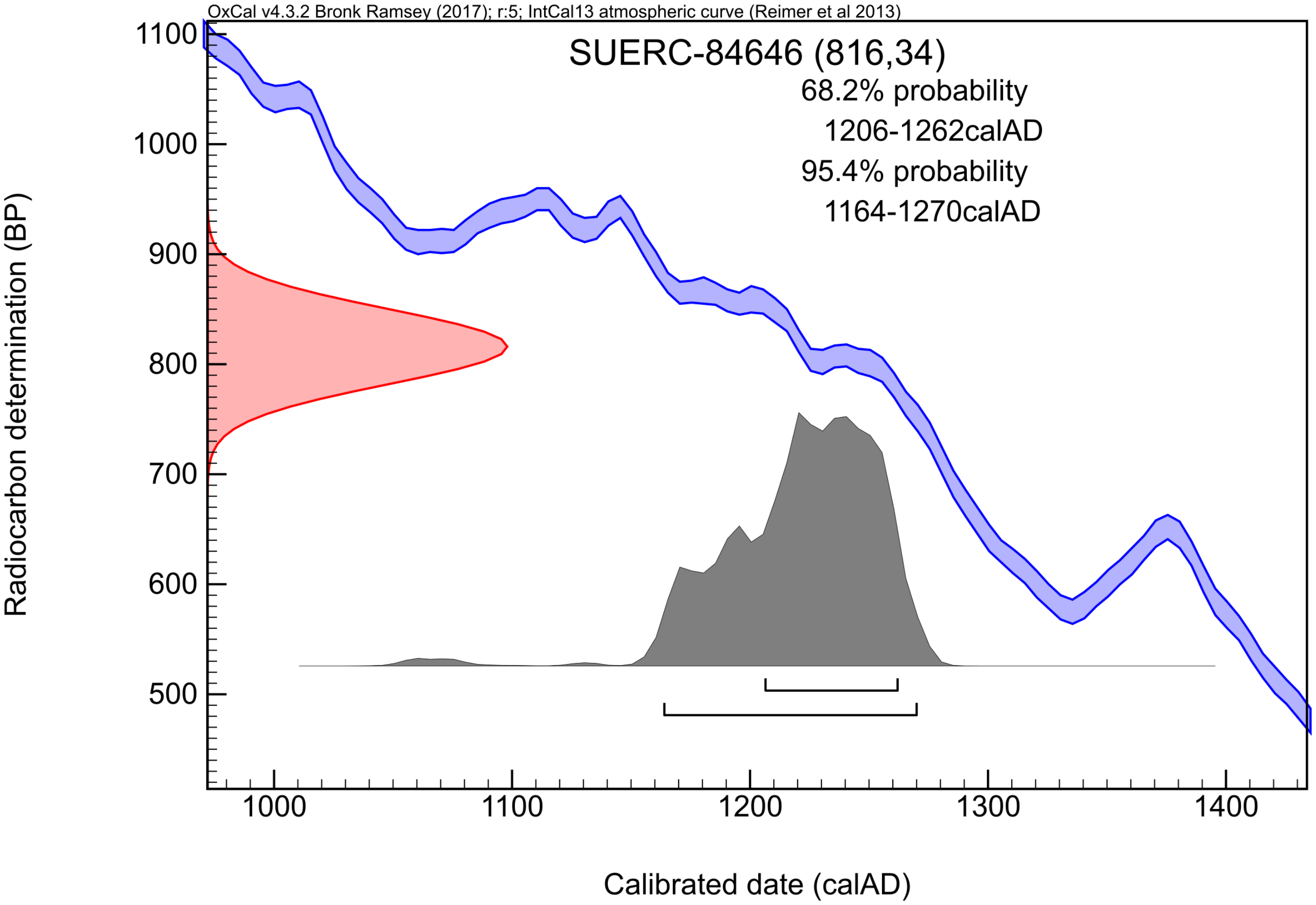

The radiocarbon age given overleaf is calibrated to the calendar timescale using the Oxford Radiocarbon Accelerator Unit calibration program OxCal 4.*

The above date ranges have been calibrated using the IntCal13 atmospheric calibration curve.†

Please contact the laboratory if you wish to discuss this further.

* Bronk Ramsey (2009) *Radiocarbon 51(1) pp.337-60*
† Reimer et al. (2013) *Radiocarbon 55(4) pp.1869-87*

Scottish Universities Environmental Research Centre
Rankine Avenue, Scottish Enterprise Technology Park, East Kilbride, Glasgow G75 0QF, Scotland, UK
Director: Professor F M Stuart Tel: +44 (0)1355 223332 Fax: +44 (0)1355 229898 www.glasgow.ac.uk/suerc

RADIOCARBON DATING CERTIFICATE
20 February 2019

**Laboratory Code**	SUERC-84647 (GU50190)
**Submitter**	Paolo Forlin<br>University of Durham<br>Department of Archaeology<br>South Road<br>DH1 3LE<br>Durham
**Site Reference**	GR-EC18
**Context Reference**	210
**Sample Reference**	C14-04
**Material**	Charred plant remain : cf. Hordeum sp.
**$\delta^{13}C$ relative to VPDB**	-25.3 ‰
**Radiocarbon Age BP**	811 ± 34

N.B. The above ^{14}C age is quoted in conventional years BP (before 1950 AD) and requires calibration to the calendar timescale. The error, expressed at the one sigma level of confidence, includes components from the counting statistics on the sample, modern reference standard and blank and the random machine error.

Samples with a SUERC coding are measured at the Scottish Universities Environmental Research Centre AMS Laboratory and should be quoted as such in any reports within the scientific literature. The laboratory GU coding should also be given in parentheses after the SUERC code.

Detailed descriptions of the methods employed by the SUERC Radiocarbon Laboratory can be found in Dunbar et al. (2016) *Radiocarbon 58(1) pp.9-23.*

For any queries relating to this certificate, the laboratory can be contacted at suerc-c14lab@glasgow.ac.uk.

Conventional age and calibration age ranges calculated by : B Tagney

Checked and signed off by : P. Naysmith

The University of Glasgow, charity number SC004401

The University of Edinburgh is a charitable body, registered in Scotland, with registration number SC005336

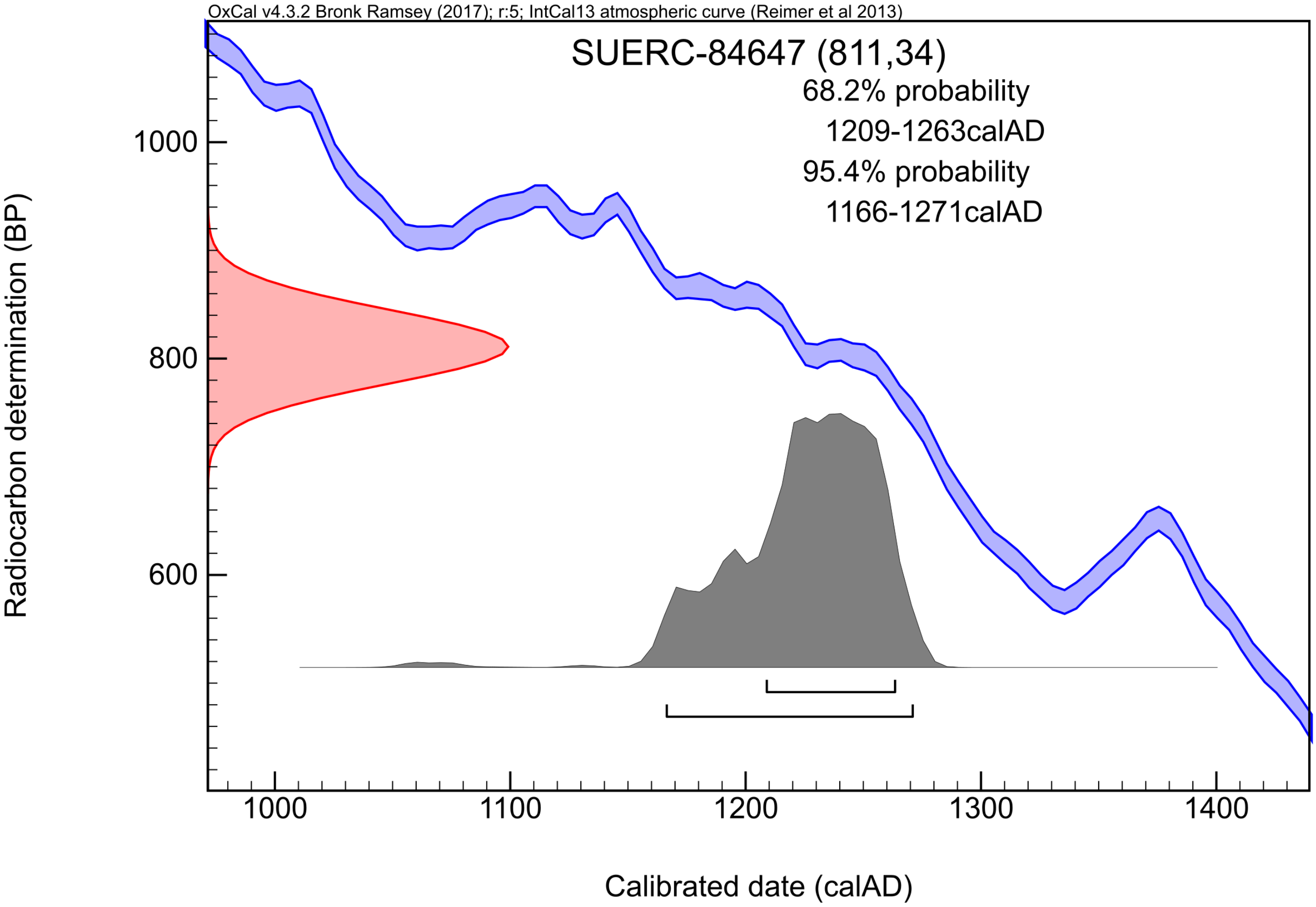

The radiocarbon age given overleaf is calibrated to the calendar timescale using the Oxford Radiocarbon Accelerator Unit calibration program OxCal 4.*

The above date ranges have been calibrated using the IntCal13 atmospheric calibration curve.†

Please contact the laboratory if you wish to discuss this further.

* Bronk Ramsey (2009) *Radiocarbon 51(1) pp.337-60*
† Reimer et al. (2013) *Radiocarbon 55(4) pp.1869-87*

Scottish Universities Environmental Research Centre
Rankine Avenue, Scottish Enterprise Technology Park, East Kilbride, Glasgow G75 0QF, Scotland, UK
Director: Professor F M Stuart Tel: +44 (0)1355 223332 Fax: +44 (0)1355 229898 www.glasgow.ac.uk/suerc

RADIOCARBON DATING CERTIFICATE
20 February 2019

**Laboratory Code**	SUERC-84648 (GU50191)
**Submitter**	Paolo Forlin<br>University of Durham<br>Department of Archaeology<br>South Road<br>DH1 3LE<br>Durham
**Site Reference**	GR-EC18
**Context Reference**	108
**Sample Reference**	C14-05
**Material**	Charred plant remain : Olea europaea L. (fragment)
**$\delta^{13}C$ relative to VPDB**	-22.6 ‰
**Radiocarbon Age BP**	841 ± 34

N.B. The above ^{14}C age is quoted in conventional years BP (before 1950 AD) and requires calibration to the calendar timescale. The error, expressed at the one sigma level of confidence, includes components from the counting statistics on the sample, modern reference standard and blank and the random machine error.

Samples with a SUERC coding are measured at the Scottish Universities Environmental Research Centre AMS Laboratory and should be quoted as such in any reports within the scientific literature. The laboratory GU coding should also be given in parentheses after the SUERC code.

Detailed descriptions of the methods employed by the SUERC Radiocarbon Laboratory can be found in Dunbar et al. (2016) *Radiocarbon 58(1) pp.9-23.*

For any queries relating to this certificate, the laboratory can be contacted at suerc-c14lab@glasgow.ac.uk.

Conventional age and calibration age ranges calculated by :

Checked and signed off by :

The University of Glasgow, charity number SC004401

The University of Edinburgh is a charitable body, registered in Scotland, with registration number SC005336

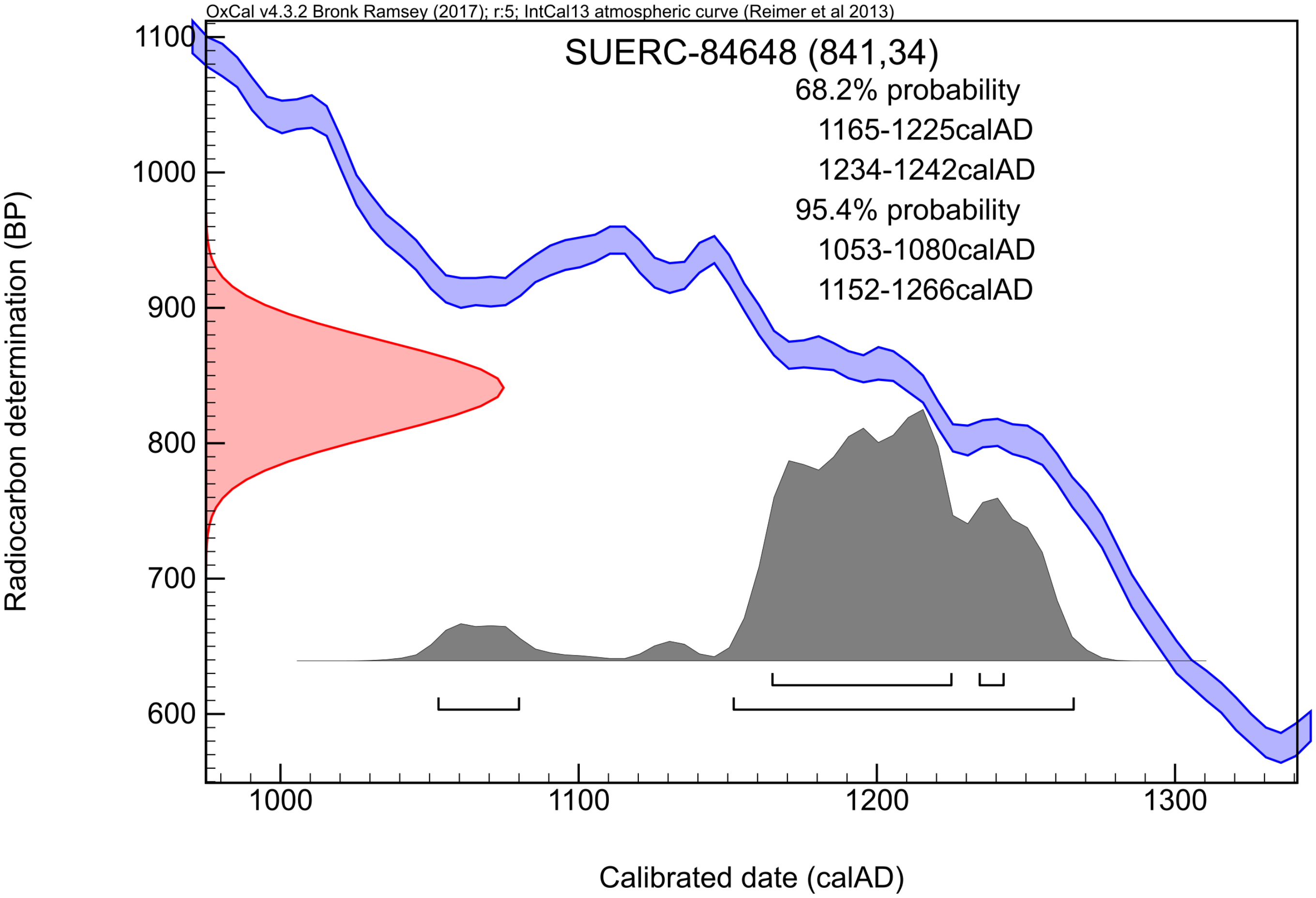

The radiocarbon age given overleaf is calibrated to the calendar timescale using the Oxford Radiocarbon Accelerator Unit calibration program OxCal 4.*

The above date ranges have been calibrated using the IntCal13 atmospheric calibration curve.†

Please contact the laboratory if you wish to discuss this further.

* Bronk Ramsey (2009) *Radiocarbon 51(1) pp.337-60*
† Reimer et al. (2013) *Radiocarbon 55(4) pp.1869-87*

Scottish Universities Environmental Research Centre
Rankine Avenue, Scottish Enterprise Technology Park, East Kilbride, Glasgow G75 0QF, Scotland, UK
Director: Professor F M Stuart Tel: +44 (0)1355 223332 Fax: +44 (0)1355 229898 www.glasgow.ac.uk/suerc

RADIOCARBON DATING CERTIFICATE
20 February 2019

Laboratory Code SUERC-84649 (GU50192)

Submitter Paolo Forlin
University of Durham
Department of Archaeology
South Road
DH1 3LE
Durham

Site Reference GR-EC18
Context Reference 108
Sample Reference C14-06

Material Charred plant remain

$\delta^{13}C$ relative to VPDB -23.9 ‰

Radiocarbon Age BP 761 ± 34

N.B. The above ^{14}C age is quoted in conventional years BP (before 1950 AD) and requires calibration to the calendar timescale. The error, expressed at the one sigma level of confidence, includes components from the counting statistics on the sample, modern reference standard and blank and the random machine error.

Samples with a SUERC coding are measured at the Scottish Universities Environmental Research Centre AMS Laboratory and should be quoted as such in any reports within the scientific literature. The laboratory GU coding should also be given in parentheses after the SUERC code.

Detailed descriptions of the methods employed by the SUERC Radiocarbon Laboratory can be found in Dunbar et al. (2016) *Radiocarbon 58(1) pp.9-23.*

For any queries relating to this certificate, the laboratory can be contacted at suerc-c14lab@glasgow.ac.uk.

Conventional age and calibration age ranges calculated by :

Checked and signed off by :

The University of Glasgow, charity number SC004401

The University of Edinburgh is a charitable body, registered in Scotland, with registration number SC005336

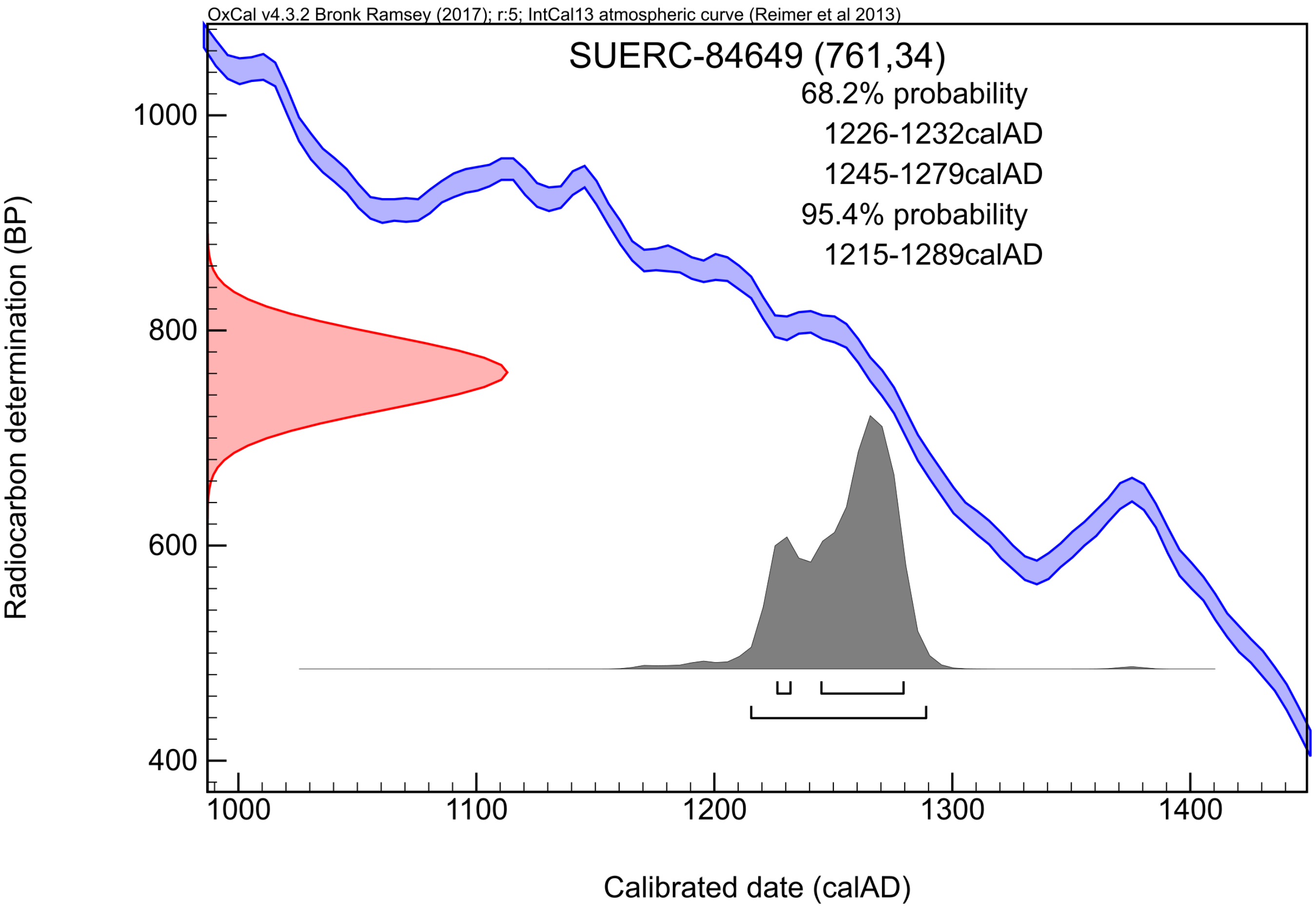

The radiocarbon age given overleaf is calibrated to the calendar timescale using the Oxford Radiocarbon Accelerator Unit calibration program OxCal 4.*

The above date ranges have been calibrated using the IntCal13 atmospheric calibration curve.†

Please contact the laboratory if you wish to discuss this further.

* Bronk Ramsey (2009) *Radiocarbon 51(1) pp.337-60*
† Reimer et al. (2013) *Radiocarbon 55(4) pp.1869-87*

Scottish Universities Environmental Research Centre
Rankine Avenue, Scottish Enterprise Technology Park, East Kilbride, Glasgow G75 0QF, Scotland, UK
Director: Professor F M Stuart Tel: +44 (0)1355 223332 Fax: +44 (0)1355 229898 www.glasgow.ac.uk/suerc

RADIOCARBON DATING CERTIFICATE
20 February 2019

**Laboratory Code**	SUERC-84650 (GU50193)
**Submitter**	Paolo Forlin<br>University of Durham<br>Department of Archaeology<br>South Road<br>DH1 3LE<br>Durham
**Site Reference**	GR-EC18
**Context Reference**	105
**Sample Reference**	C14-07
**Material**	Charred plant remain : Fruit fragment cf. Vitis vinifera
**$\delta^{13}C$ relative to VPDB**	-23.8 ‰
**Radiocarbon Age BP**	784 ± 34

N.B. The above ^{14}C age is quoted in conventional years BP (before 1950 AD) and requires calibration to the calendar timescale. The error, expressed at the one sigma level of confidence, includes components from the counting statistics on the sample, modern reference standard and blank and the random machine error.

Samples with a SUERC coding are measured at the Scottish Universities Environmental Research Centre AMS Laboratory and should be quoted as such in any reports within the scientific literature. The laboratory GU coding should also be given in parentheses after the SUERC code.

Detailed descriptions of the methods employed by the SUERC Radiocarbon Laboratory can be found in Dunbar et al. (2016) *Radiocarbon 58(1) pp.9-23.*

For any queries relating to this certificate, the laboratory can be contacted at suerc-c14lab@glasgow.ac.uk.

Conventional age and calibration age ranges calculated by :

Checked and signed off by :

The University of Glasgow, charity number SC004401

The University of Edinburgh is a charitable body, registered in Scotland, with registration number SC005336

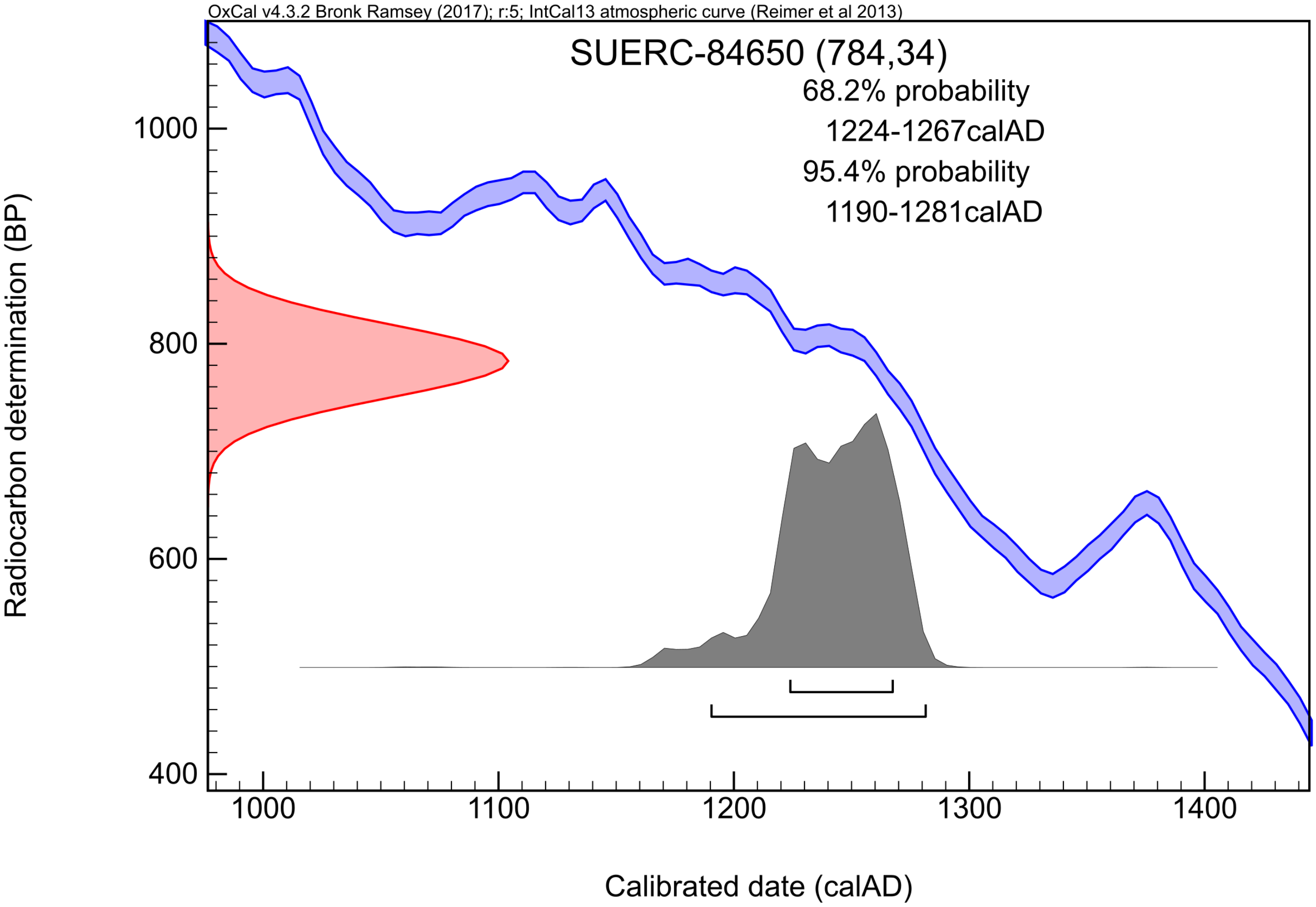

The radiocarbon age given overleaf is calibrated to the calendar timescale using the Oxford Radiocarbon Accelerator Unit calibration program OxCal 4.*

The above date ranges have been calibrated using the IntCal13 atmospheric calibration curve.†

Please contact the laboratory if you wish to discuss this further.

* Bronk Ramsey (2009) *Radiocarbon 51(1) pp.337-60*
† Reimer et al. (2013) *Radiocarbon 55(4) pp.1869-87*

Scottish Universities Environmental Research Centre
Rankine Avenue, Scottish Enterprise Technology Park, East Kilbride, Glasgow G75 0QF, Scotland, UK
Director: Professor F M Stuart Tel: +44 (0)1355 223332 Fax: +44 (0)1355 229898 www.glasgow.ac.uk/suerc

RADIOCARBON DATING CERTIFICATE

20 February 2019

**Laboratory Code**	SUERC-84651 (GU50194)
**Submitter**	Paolo Forlin<br>University of Durham<br>Department of Archaeology<br>South Road<br>DH1 3LE<br>Durham
**Site Reference**	GR-EC18
**Context Reference**	3011
**Sample Reference**	C14-08
**Material**	Charcoal : Fabaceae (roundwood)
**$\delta^{13}C$ relative to VPDB**	-24.5 ‰
**Radiocarbon Age BP**	789 ± 34

N.B. The above ^{14}C age is quoted in conventional years BP (before 1950 AD) and requires calibration to the calendar timescale. The error, expressed at the one sigma level of confidence, includes components from the counting statistics on the sample, modern reference standard and blank and the random machine error.

Samples with a SUERC coding are measured at the Scottish Universities Environmental Research Centre AMS Laboratory and should be quoted as such in any reports within the scientific literature. The laboratory GU coding should also be given in parentheses after the SUERC code.

Detailed descriptions of the methods employed by the SUERC Radiocarbon Laboratory can be found in Dunbar et al. (2016) *Radiocarbon 58(1) pp.9-23.*

For any queries relating to this certificate, the laboratory can be contacted at suerc-c14lab@glasgow.ac.uk.

Conventional age and calibration age ranges calculated by : [signature]

Checked and signed off by : P. Naysmith

The University of Glasgow, charity number SC004401

The University of Edinburgh is a charitable body, registered in Scotland, with registration number SC005336

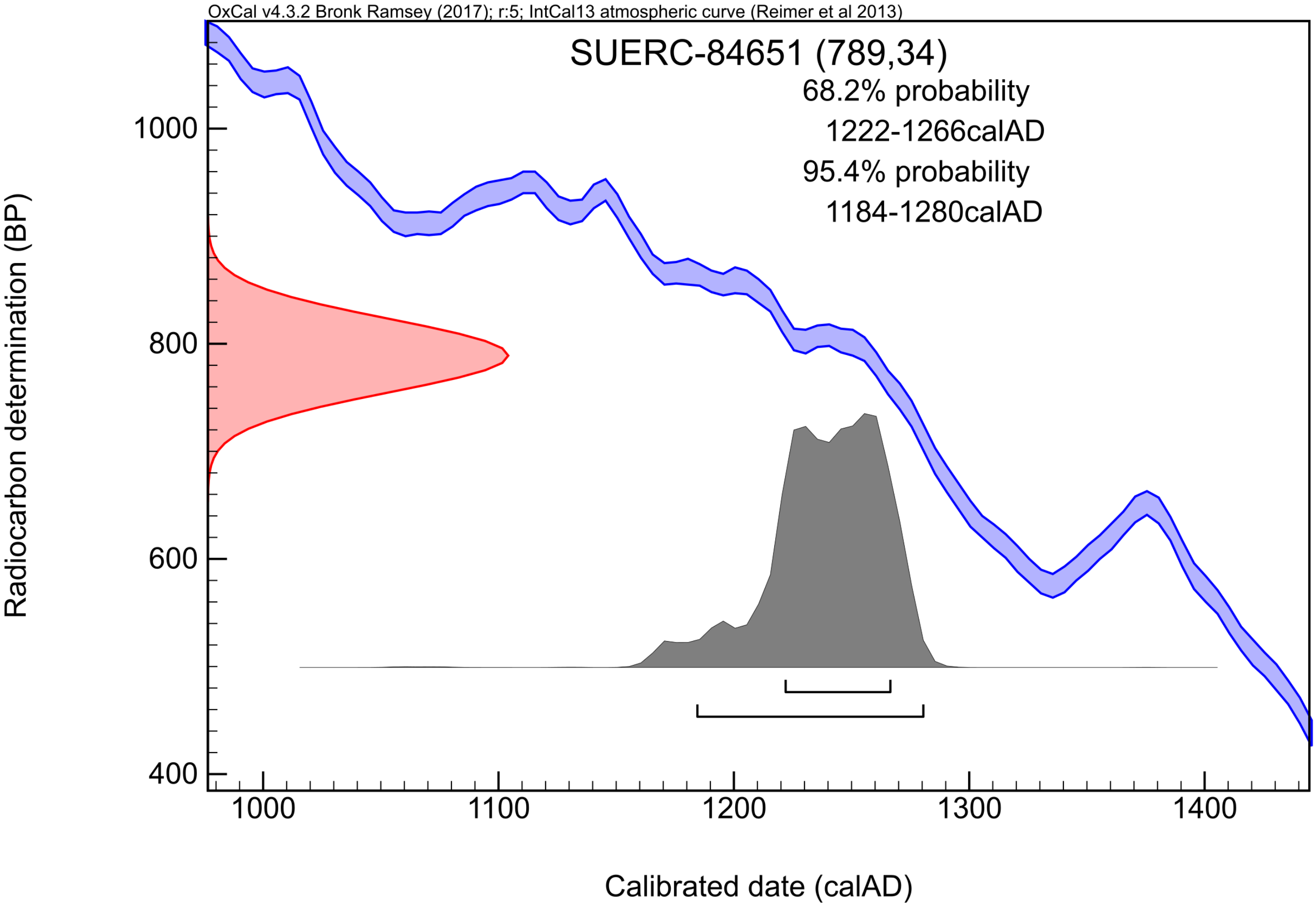

The radiocarbon age given overleaf is calibrated to the calendar timescale using the Oxford Radiocarbon Accelerator Unit calibration program OxCal 4.*

The above date ranges have been calibrated using the IntCal13 atmospheric calibration curve.†

Please contact the laboratory if you wish to discuss this further.

* Bronk Ramsey (2009) *Radiocarbon 51(1) pp.337-60*
† Reimer et al. (2013) *Radiocarbon 55(4) pp.1869-87*
