# Supplementary material for: Recovering a lost seismic disaster. The destruction of El Castillejo and the discovery of the earliest historic earthquake affecting the Granada region (Spain)
Source: PLoS One. 2024 Apr 17;19(4):e0300549. doi: 10.1371/journal.pone.0300549 (PMC11023601; doi:10.1371/journal.pone.0300549)
Supplement: S2 File — (DOCX) [file pone.0300549.s003.docx]

OxCal code

Options()

 {

  Resolution=1;

 };

 Plot()

 {

  Combine("Trench 1")

  {

   R_Date("C14-05", 841, 34);

   R_Date("C14-06", 761, 34);

   R_Date("C14-07", 784, 34);

  };

  Combine("Trench 2")

  {

   R_Date("C14-01", 782, 34);

   R_Date("C14-02", 800, 34);

   R_Date("C14-03", 816, 34);

   R_Date("C14-04", 811, 34);

  };

  Combine("Trench 3")

  {

   R_Date("C14-08", 789, 34);

  };

  Combine("all")

  {

   R_Date("C14-01", 782, 34);

   R_Date("C14-02", 800, 34);

   R_Date("C14-03", 816, 34);

   R_Date("C14-04", 811, 34);

   R_Date("C14-05", 841, 34);

   R_Date("C14-06", 761, 34);

   R_Date("C14-07", 784, 34);

   R_Date("C14-08", 789, 34);

  };

 };
