# Supplementary material for: Recovering a lost seismic disaster. The destruction of El Castillejo and the discovery of the earliest historic earthquake affecting the Granada region (Spain)
Source: PLoS One. 2024 Apr 17;19(4):e0300549. doi: 10.1371/journal.pone.0300549 (PMC11023601; doi:10.1371/journal.pone.0300549)

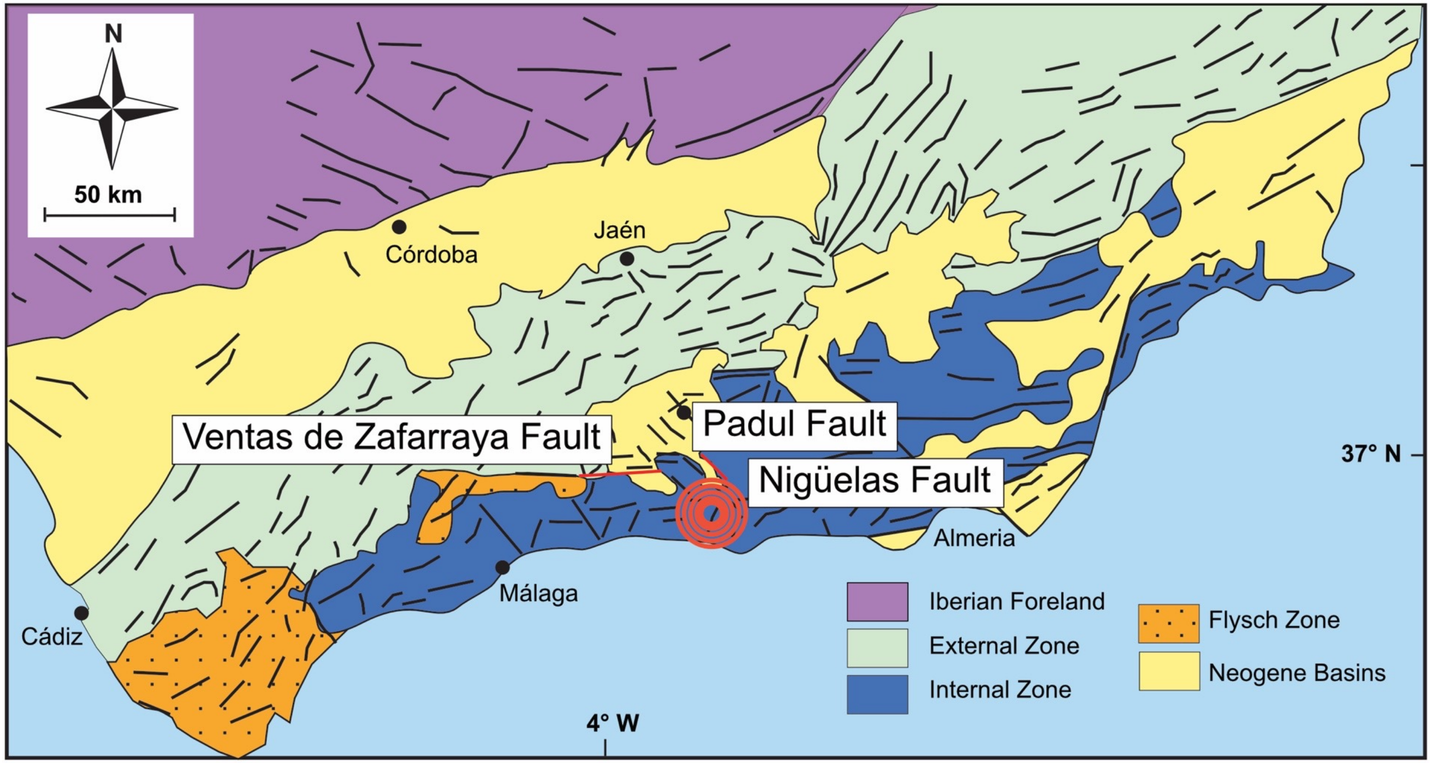

Seismogenic Faults in the study area, concentric red circles – El Castillejo

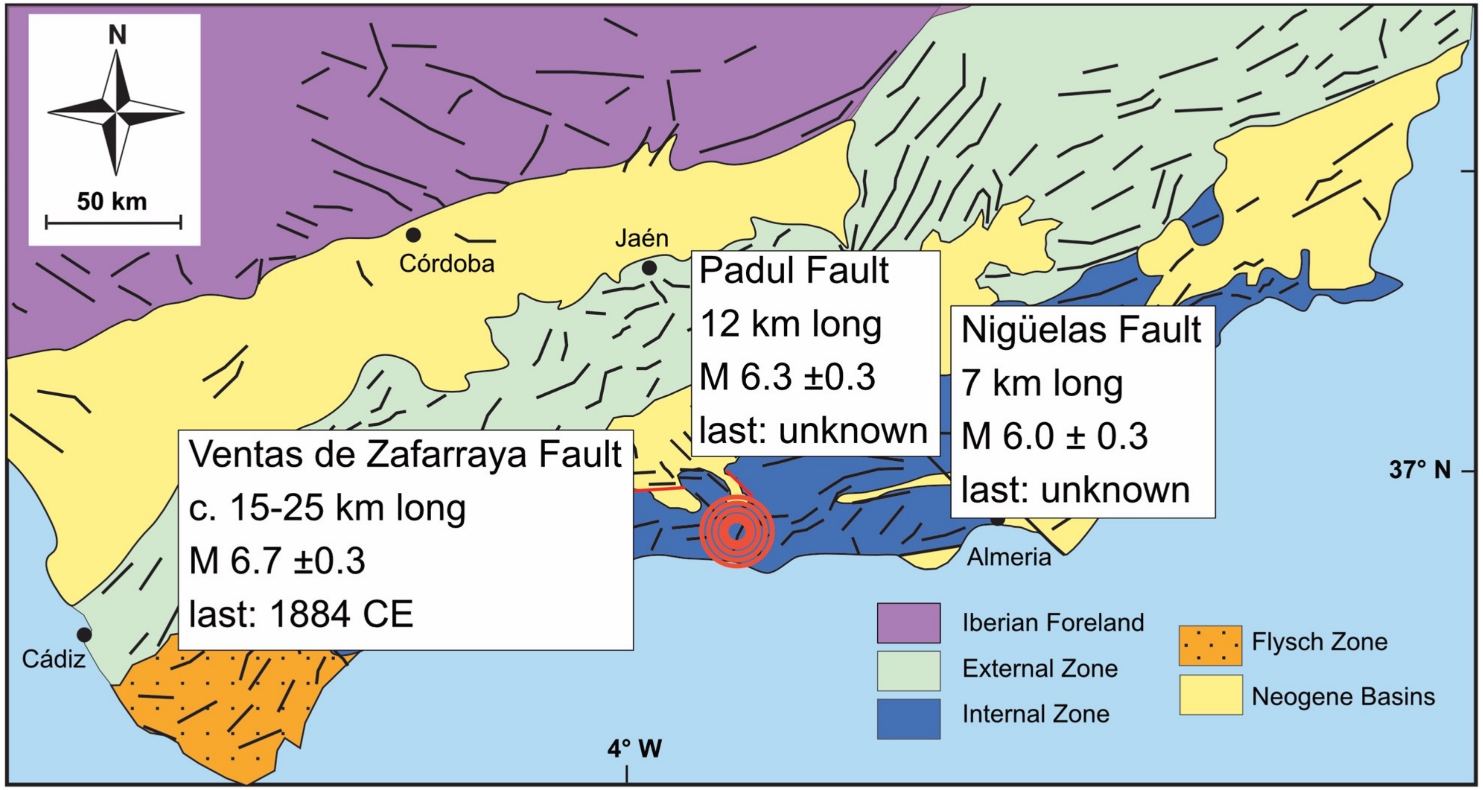

Seismogenic Faults in the study area, concentric red circles – El Castillejo-
Length, possible M (capable for), and last eq

Scenario: 1884; Earthquake in Ventas de Zaffaraya Fault
magnitude: 6.7, depth: 10 km, maximum intensity: 8.7

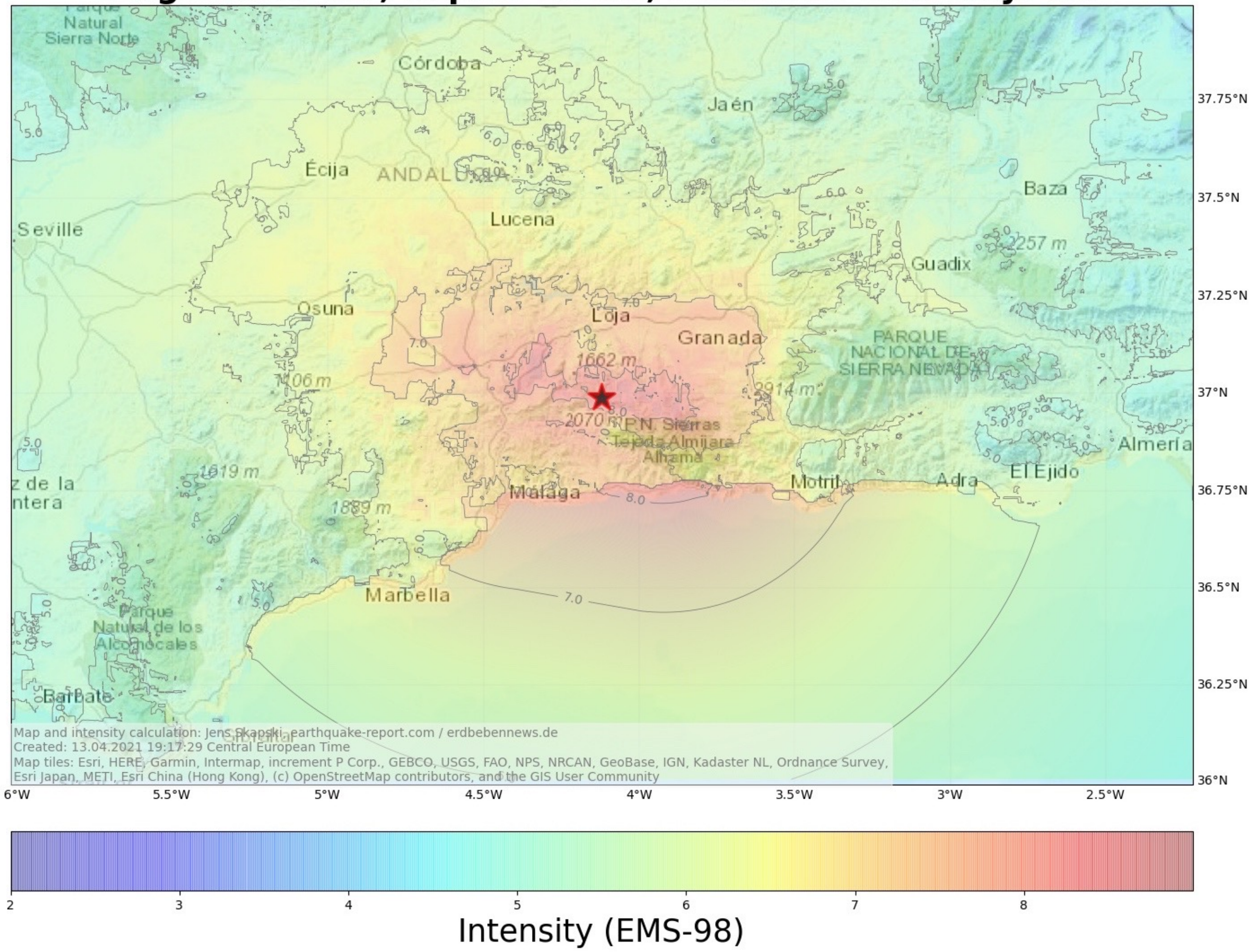

Scenario: 3000 BP; Earthquake in Niguelas Fault
magnitude: 6.0, depth: 6 km, maximum intensity: 8.1

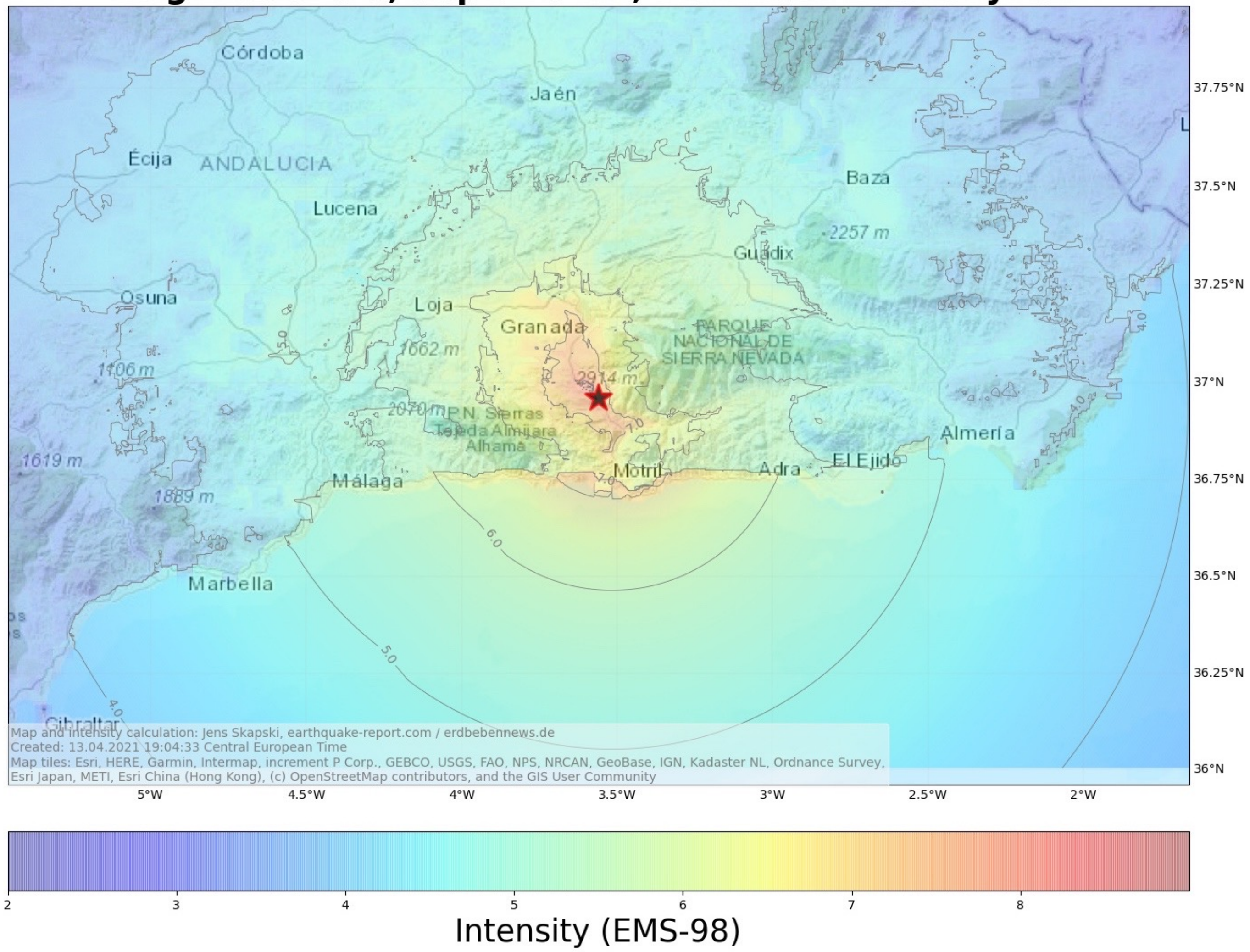

Scenario: 3000 BP; Earthquake in Padul Fault
magnitude: 6.3, depth: 8 km, maximum intensity: 8.5

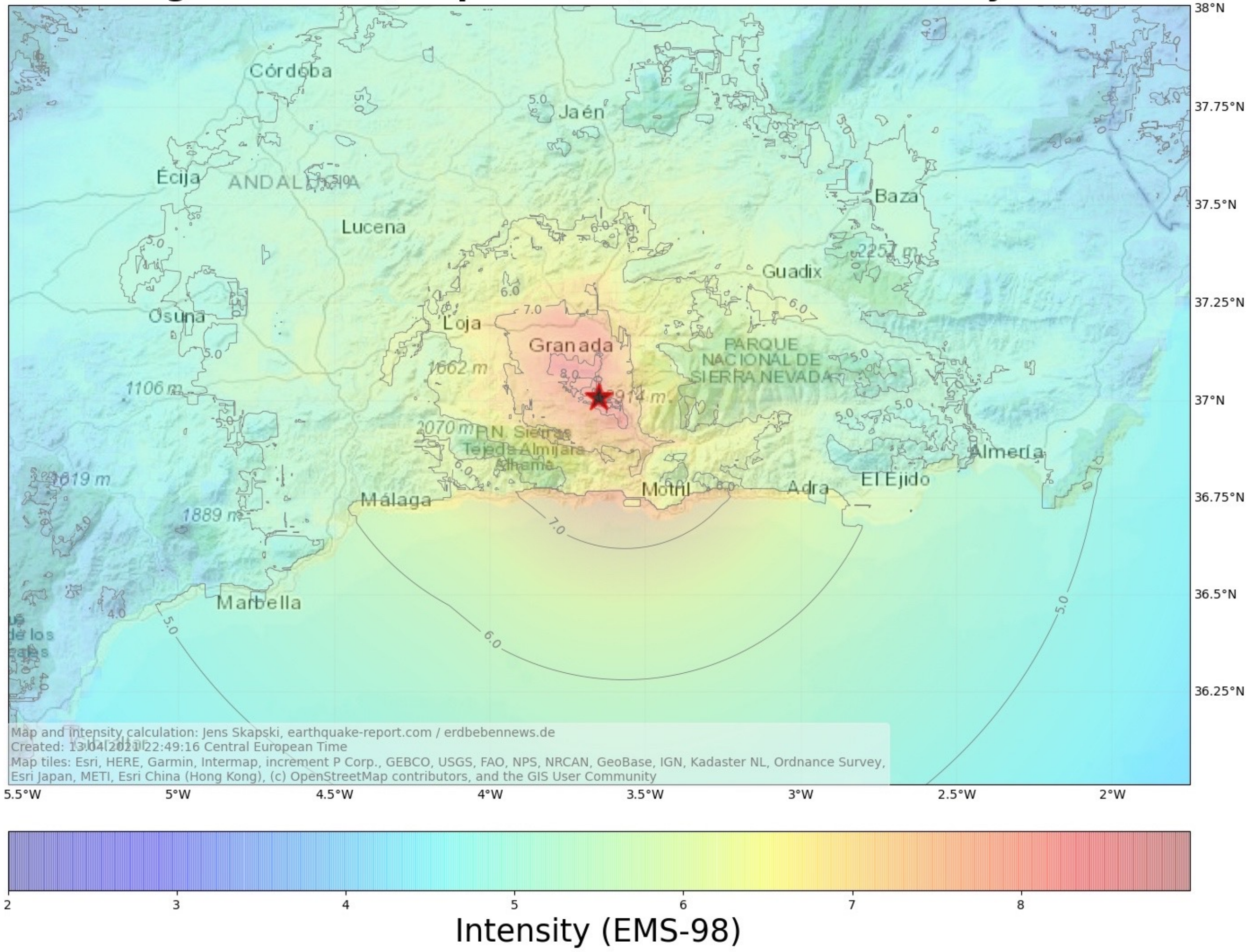

Scenario: 1240; Earthquake in El Castillejo
magnitude: 6.0, depth: 10 km, maximum intensity: 8.6

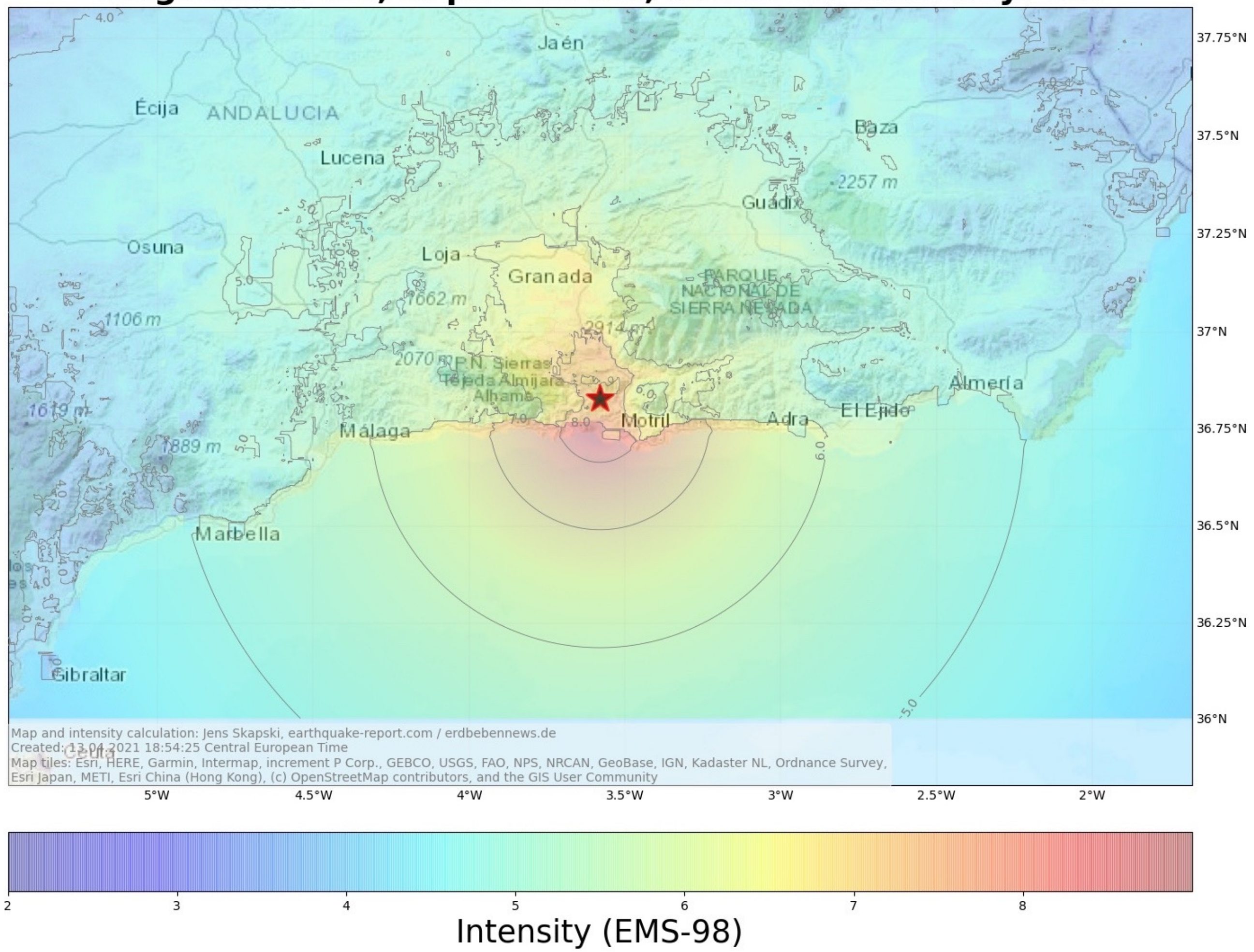

Supplement: S3 File — (PDF) [file pone.0300549.s004.pdf]
