# Supplementary material for: Recovering a lost seismic disaster. The destruction of El Castillejo and the discovery of the earliest historic earthquake affecting the Granada region (Spain)
Source: PLoS One. 2024 Apr 17;19(4):e0300549. doi: 10.1371/journal.pone.0300549 (PMC11023601; doi:10.1371/journal.pone.0300549)
Supplement: S5 File — (DOCX) [file pone.0300549.s006.docx]

**Supplementary OSL**

*Sample locations*

The sediment samples were taken from three locations including: a) in Trench 1, the upper deposits of layer 117 that comprised a terrace fill; b) in Trench 3, layer 305 immediately underlying the destruction deposits (context 304) and the lower part of the layer 315, both of which were located within a sequence of laminated sediments interpreted to be associated with modification of dwellings. The brick samples were obtained from two locations: a) an exposed fragment of a single brick (sample 438-2) incorporated in the rammed earth fabric of the south wall forming the standing remains of Building 10; b) a brick fragment (sample 438-5) recovered from the basal deposits of context 119 in Trench 1 that contained disaggregated rammed earth.

*Experimental*

Initial testing of the OSL properties of the prepared measurement samples revealed that none of the sediment samples contained quartz producing measurable OSL signals, and consequently no further measurements were performed with the sediment samples. A modified single aliquot regeneration (SAR) procedure was applied to determine the paleodose (P), developed for the dating of bricks (Bailiff, 2007); the weighted average value of P was obtained for each sample from the testing of multiple single aliquots of quartz grains.

The total dose rate, D_r_, to the quartz grains was assessed by taking into account the primary contributions from beta and gamma radiation emitted by lithogenic radionuclides within the brick fabric and the surrounding environmental materials, and cosmic radiation using the calculations of (Prescott and Hutton, 1988). Infinite medium dose rates were calculated using the specific activities of ^238^U, ^232^Th and ^40^K in brick, rammed earth and soils forming the surrounding ground surface, measured using a calibrated high-resolution gamma spectrometer, and subsequently converted to dose rate using factors calculated previously (Bailiff et al., 2014) that are similar to those given in Guérin et al. (2011). Adjustments to the calculated infinite medium gamma dose rates at the sample positions of 438-2 and 438-5 were made for the combination of geometry and material composition using radiation transport simulations. Adjustment to the infinite medium beta dose rate for the effect of grain size attenuation was made using our own radiation transport calculations that produced results similar to those obtained by Brennan (2003).

As discussed in the main text, in the case of brick sample 438-2 incorporated in the rammed earth wall of Building 10, there is the question of whether the brick had been unused or recycled from an older brick structure. if the latter (Use History B), the gamma dose rate during its first use within the structure would have been higher than that present in the rammed earth wall. To illustrate the effect of History B, the average gamma dose rate was adjusted to account for the emplacement of the brick in a wall of similar brick composition for a hypothetically selected period of 200 years, after which it was incorporated as rubble in the rammed earth wall.

*References*

Bailiff, I.K. 2007 Methodological developments in the luminescence dating of brick from English late medieval and post medieval buildings, Archaeometry, 49, 827-851.

Bailiff, I.K., French, C.A., Scarre, C.J., 2014. Application of luminescence dating and geomorphological analysis to the study of landscape evolution, settlement and climate change on the Channel Island of Herm. Journal of Archaeological Science 41, 890-903.

Brennan, B.J., 2003. Beta doses to spherical grains. Radiation Measurements 37, 299-303.

Guérin, G., Mercier, N., Adamiec, G., 2011. Dose-rate conversion factors: update, Ancient TL 29, 5-8.

Prescott, J.R., Hutton, J.T., 1988. Cosmic ray and gamma ray dosimetry for TL and ESR. Radiation Measurements 14, 223-227.
