# Supplementary material for: Recovering a lost seismic disaster. The destruction of El Castillejo and the discovery of the earliest historic earthquake affecting the Granada region (Spain)
Source: PLoS One. 2024 Apr 17;19(4):e0300549. doi: 10.1371/journal.pone.0300549 (PMC11023601; doi:10.1371/journal.pone.0300549)
Supplement: Attachment — Submitted filename: Response to reviewers.docx [file pone.0300549.s007.docx]

**Response to reviewers**

**Reviewer 1**

**Questions about OSL dating**

1.	In the present format I do not understand the meaning of ages A and B of sample 438-2, and their relation with sample 438-5 (table 2 and lines 415-427. Please, explain better samples A and B. What is the meaning/interpretation of authors. After reading the text I understand that A represents a brick from a construction moment.

*Reply:	Ages A and B are the ages calculated for the same sample (438-2) using different values of the dose rate to account for two potentially different use histories. In calculating the age, A, the dose rate was assessed assuming that the brick was manufactured shortly before its incorporation in the rammed earth wall. However, in the case of a brick building we also need to consider the possibility that the brick was taken from a demolished building and reused as rubble in the wall. If so, the brick is likely to have been located in a different environment for part of its history of use. To account for this possibility (B), the components of the total dose rate which are affected (gamma and cosmic), amounting to roughly a quarter of the total, were calculated assuming (hypothetically) that the brick was located within a wall constructed with similar brick for a period of 200 years. Hence two age estimates, A and B, were calculated for the same brick. The main purpose of producing two ages is to flag the assumption made regarding history of use of ceramic building materials following manufacture when calculating the luminescence age and to provide an indication of the magnitude of the effect. To clarify this aspect for the reader the main text and Appendix 5 have been amended.*

2. They also provide the overdispersion value for two of the three samples. Why not the other one?

*Reply:	As clarified above, two samples were tested, not three.*

3 They refer to the overdispersion value. Do they mean the overdispersion of the Central Age Model (Galbraith et al., 1999)? If they are bricks, what does the overdispersion provide? This parameter is used to check complete bleaching of sediments, but bricks were fired. What it does provide?

*Reply:	The calculation of the weighted average of the log De values and overdispersion parameter, σ, provide parameter values that are also relevant to thermally reset samples. In the case of sediment, the overdispersion is usually dominated by the effects of non-uniform optical bleaching, which is not an issue for thoroughly heated ceramic samples, although potentially an issue where thermal resetting was not complete. Heterogeneity in the beta radiation field is another potential source of overdispersion for both types of sample, and in the case of thermally reset materials this consequently includes a measure of the extent of beta heterogeneity. Although the OSL measurements reported here were not single grain, the number of dominant bright grains in the small single aliquots used is likely to be less than four. For heated samples, a relatively low value of overdispersion suggests a radiometrically relatively uniform ceramic matrix.*

4 In the Supplementary OSL material, the authors refer to sediment samples from three locations. I am, unable to see the results.

*Reply: As indicated in the main text (lines 165-166), the quartz coarse grains extracted from the sediment samples did not produce measurable OSL and consequently there were no results to present. We have taken the opportunity to provide more detailed supporting information in Appendix 5, including a reiteration of the finding of very weak OSL with the quartz extracted from the sediment.*

**Questions about the chronological model.**

5. The authors used Oxcal and radiocarbon ages to get a more precise age. I agree, but they have other ages that could be used to improve the model. I refer to OSL ages.Why the authors did not used such ages in the model?

*Reply* :	*We did not include the OSL date in our original model since we wanted to rely on a consistent dataset of dates extracted from organic samples of short-lived plants. The OSL dated brick presented* some issue relating the chronology, *either expressing manufacturing or reheating. In any case, based on R1 suggestion, we calculated a new Bayesian model including the OSL date and the result is (almost) identical to the one obatined with the only radiocarbon dates with only a 1-yr shift (again, a time interval of ca. 40 years between CE 1225-1267 at 95.4% probability). However, we prefer to derive the estimated range for the event from the radiocarbon dates derived from short-lived plants recovered in primary archaeological contexts. Nevertheless, given the consistency of the OSL date with the c14 dates, we decided to include the sample 438-5 in the plot of the radiocarbon dates*.

**Reviewer 2**

**Questions about previous excavation and seismic damage:**

1. In my opinion, it is needed a more detail explanation about the "extensive" excavation carry out in the 80s: surface, significance of the sample.

*Reply* :	*We included an estimate of the sample excavated by previous research (lines 197-200)which amounts to 40% of the site’s surface.*

2. It is strange that cracks are not attested in the east area of the site, and there are relevant differences in the external wall. What is more the distribution of the different deformations attested in figure 3 seems follow some kind of pattern.

*Reply* :	*Cracks are less attested in the eastern area because this part of the site was only partially excavated (see plan in Fig 2) compared to the western part which was fully investigated. A large amount of ruined but still standing walls lie still buried and therefore the identification of cracks on architectural remains is particularly difficult in this area. However, as demonstrated by our research (see Trench 2), new cracks have been found on walls originally covered by debris. The external are also poorly preserved and cracks here are absent. The gate was rebuilt and here any seismic damage was removed in occasion of the rebuilding efforts. Finally, it is difficult to observe a pattern in the orientation of the deformations observed at El Castillejo. Shapes, orientations and distribution of the damage seem depending on the orientation of the structures, interaction with other structures, relationships with the local (artificially modified) profile of the bedrock.*

**Questions about the reoccupation of the site:**

3. I would like to know more about the reuse of the site after the seism event. Is there a hiatus? The reconstruction affects all the site? Is attested some change in the building techniques or materials? I believe that the cultural consequences of the event are not fully explored.

*Reply* :	*This is clearly a critical aspect in the ‘history’ of the site, even if -as it emerges clearly- this was not the main objective of this work. We think we have better clarified ‘where’ and ‘how’ the reconstruction took place, and highlighted how difficult is, as of the current state of knowledge, to understand ‘when’ (211-220). We have then briefly reflected on the cultural impact of the event (509-517) and wished for the prosecution of research and study on the archaeological record of this important site. As stated above, the goal of this project was to assess and date the destrcutvie event that affect the settlement marking the end of its phase one.*

4. In a more general way, is this the first time that this approach has been used?

*Reply* : *We think that this was the first time that an archaeological project was carried out with the purposes of verifying the occurrence and chronology of an unknown seismic event by combining archaeological and architectural analysis with absolute dating.*
