# Supplementary material for: Recovering a lost seismic disaster. The destruction of El Castillejo and the discovery of the earliest historic earthquake affecting the Granada region (Spain)
Source: PLoS One. 2024 Apr 17;19(4):e0300549. doi: 10.1371/journal.pone.0300549 (PMC11023601; doi:10.1371/journal.pone.0300549)
Supplement: Attachment — Submitted filename: Response to reviewers.docx [file pone.0300549.s008.docx]

Response to reviewers

We sincerely thank the reviewers #3 and #4 for their valuable and constructive comments.

We believe we have addressed the two points raised by reviewer #3.

Firstly, we have included the reference of a very recent damage survey compiled in the immediate aftermath of the Oukaïmedene Morocco Earthquake which shows damage to rammed earth structures that look totally consistent with those observed at El Castillejo (line 473).

Secondly, we have corrected the estimated number of the books burned in the fire of Plaza Bib-Rambla in Granada. We have also included the suggested reference of the ‘*Memorial de la vida de Fray Francisco Jiménez de Cisneros*’. This comment was particularly significant as it allowed us to correct an exaggerated figure drawn from less accurate studies.

Again, we are truly grateful to the reviewers for their positive feedback and highly relevant suggestions.
